# Cost-effectiveness analysis of combining lenalidomide with R-CHOP for treating diffuse large B-cell lymphoma in China

**DOI:** 10.3389/fphar.2024.1412743

**Published:** 2024-12-16

**Authors:** Rongqi Li, Yuhan Zeng, Yizhang Chen, Zhongjiang Ye, Chuang Chen, Jianhui Yang, Jing Fu, Tao Zhou, Danna Jiang, Sunting Qin, Haige Ye, Ziye Zhou, Xiuhua Zhang

**Affiliations:** ^1^ Department of Pharmacy, The First Affiliated Hospital of Wenzhou Medical University, Wenzhou, Zhejiang, China; ^2^ School of Pharmaceutical Sciences, Wenzhou Medical University, Wenzhou, Zhejiang, China; ^3^ Department of hematology, The First Affiliated Hospital of Wenzhou Medical University, Wenzhou, Zhejiang, China; ^4^ Clinical Research Center, The First Affiliated Hospital of Wenzhou Medical University, Wenzhou, Zhejiang, China; ^5^ Key Laboratory of Intelligent Treatment and Life Support for Critical Diseases of Zhejiang Province, Wenzhou, Zhejiang, China

**Keywords:** lenalidomide, R-CHOP, cost-effectiveness, pharmacoeconomic, diffuse large B-cell lymphoma

## Abstract

**Background:**

Lenalidomide is a thalidomide analog that has immunomodulatory and anti-angiogenic properties. The ECOC-ACRIN E1412 Phase II trial demonstrated that lenalidomide, when combined with rituximab, cyclophosphamide, doxorubicin, vincristine, and prednisone (R-CHOP), extended survival in diffuse large B-cell lymphoma (DLBCL) patients. This study aimed to evaluate the cost-effectiveness of combining lenalidomide with R-CHOP (R2-CHOP) versus R-CHOP alone as the initial treatment for DLBCL from the perspective of the Chinese healthcare system.

**Methods:**

We developed a 5-year partitioned survival model to compare the cost-effectiveness of R2-CHOP versus R-CHOP alone. The clinical data came from the ECOG-ACRIN E1412 clinical trial. The costs of drugs and examinations were obtained from publicly available Chinese medical databases and literatures. Model robustness was assessed by sensitivity analysis and scenario analysis. And subgroup analysis was also performed. Key outcomes include total cost, quality-adjusted life years, and the incremental cost-effectiveness ratio (ICER).

**Results:**

Over a 5-year time horizon, the basic analysis results of the partitioned survival model showed that the ICER of $35,159.06 per QALY for R2-CHOP compared to R-CHOP. Deterministic sensitivity analysis revealed that the price of lenalidomide is the main factor affecting cost-effectiveness. Probabilistic sensitivity analysis indicated a 67.9% chance of lenalidomide plus R-CHOP being cost-effective at the willingness-to-pay threshold, compared to R-CHOP alone. Scenario analysis showed R2-CHOP scenarios to be cost-effective for 10–30 years. And subgroup analysis showed that treating activated B cell-like type DLBCL with R2-CHOP was more cost-effective.

**Conclusion:**

In the Chinese healthcare system, R2-CHOP is a cost-effective approach for DLBCL compared to R-CHOP, but the costs of lenalidomide and rituximab warrant attention.

## Introduction

Diffuse large B-cell lymphoma (DLBCL) is the most common subtype of non-Hodgkin lymphoma (Chiappella et al., 2017). Over 60% of patients are cured by the standard treatment regimen of rituximab combined with cyclophosphamide, doxorubicin, vincristine, and prednisone (R-CHOP) (Spinner and Advani, 2022). Following the introduction of R-CHOP, subsequent clinical trials aimed to enhance its efficacy by methods such as drug addition and dose intensification (Morrison, 2021). Lenalidomide is a thalidomide analog that has immunomodulatory and anti-angiogenic properties, including modifying cytokine production, activating T cells, and boosting natural killer cell function (Segler and Tsimberidou, 2012). Several domestic and international studies have demonstrated that adding lenalidomide to DLBCL treatment improves response rates and progression-free survival (PFS), albeit with increased hematological adverse events (Castellino et al., 2018; Desai et al., 2021; Liu et al., 2023). The ECOG-ACRIN E1412 randomized phase II study found that the addition of future lenalidomide to the R-CHOP regimen improved outcomes for newly diagnosed DLBCL patients (Nowakowski et al., 2021). Although the average cost per DLBCL patient is not as high as other cancers, the total cost of DLBCL treatment still imposes a significant financial burden on patients and the healthcare system (Harkins et al., 2019). In recent years, clinical research has focused on identifying an initial treatment plan for DLBCL that improves its efficacy, safety, and cost-effectiveness. Although previous trials have demonstrated the efficacy of R2-CHOP in the treatment of DLBCL, there is no relevant literature on the pharmacoeconomic of this regimen, and the present study is designed to fill this gap in knowledge. Our goal is to investigate the cost-effectiveness of combining lenalidomide with R-CHOP (R2-CHOP) for DLBCL treatment.

## Materials and methods

### Model construction

**FIGURE 1:**
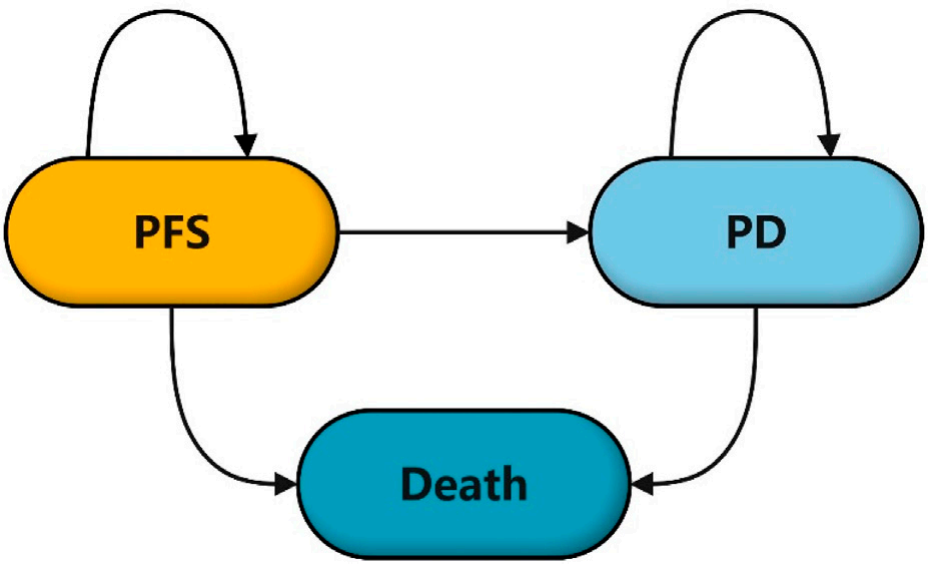
Structure diagram of partition survival model.

This study adhered to the CHEERS reporting guidelines (Don et al., 2022), and we have included a CHEERS report checklist in Supplementary Table S1. We developed a partitioned survival model to assess the medical costs and effectiveness of combining lenalidomide with R-CHOP in treating DLBCL. The partitioned survival model was constructed using Treeage Pro 2022 software. The model comprises three mutually exclusive health states: PFS, progressive disease (PD), and Death. The time horizon is set at 5 years to better reflect the actual situation. One prognostic study showed a 5-year overall survival (OS) in 64.1% DLBCL patients after treatment (Han et al., 2019). In addition, the survival curves from the ECOG-ACRIN E1412 study showed that more than half of patients with DLBCL were expected to survive beyond 5 years. The cycle length corresponds to one treatment cycle of 21 days, as shown in Figure 1.

Aiming to provide evidence on the resource costs and patient benefits of various protocols, we conducted this analysis from the perspective of the Chinese healthcare system. The main outputs of the model include cost, quality-adjusted life years (QALYs), and the incremental cost-effectiveness ratio (ICER). A 5% discount rate will be applied to the cost of the simulation results (Gan et al., 2023). According to the National Bureau of Statistics, China’s 2023 *per capita* GDP in will be ¥ 89,358 ($12,410.83). (https://www.stats.gov.cn/sj/zxfb/202402/t20240228_1947915.html). The exchange rate from China to the US is 1 USD ≈7.20 RMB. China’s willingness-to-pay (WTP) threshold is based on three times China’s GDP *per capita*. Therefore, this study set the WTP threshold at $37,232.5 per QALY.

### Clinical data

The target population is newly diagnosed, untreated, histologically proven DLBCL adult (age ≥18 years) patients with stage II bulky (measurable tumor size >10 cm) to IV. The clinical trial included 280 patients (145 patients received R2-CHOP treatment and 135 patients received R-CHOP treatment), which also included 94 activated B cell-like (ABC) type of DLBCL. In the R-CHOP group, patients received rituximab (375 mg/m^2^), cyclophosphamide (750 mg/m^2^), doxorubicin (50 mg/m^2^), and oncovin (1.4 mg/m^2^) on the first day of each cycle, plus prednisone (100 mg/m^2^) daily from day 1–5 of each cycle. In the R2-CHOP group, patients received 25 mg lenalidomide daily from day 1–10 of each cycle, in addition to the R-CHOP regimen. Assuming a typical patient weighs 65 kg and has a height of 1.64 m, this results in a body surface area of 1.72 m^2^ (Wu et al., 2011). When the PD occur, first-line treatment is discontinued, and patients start receiving second-line therapy. Following the B-lymphocyte clinical practice guidelines by the National Comprehensive Cancer Network in the US (National Comprehensive Cancer Network, 2022), the model assumes the second-line regimen received by both groups of patients with PD is the R-DHAP regimen (rituximab, cisplatin, dexamethasone, and high-dose cytarabine), and that the costs and utilities of the second-line are taken from the literature, as detailed in Table 1.

**TABLE 1:** Model parameters and distribution.

Parameter	Range				
	Baseline	Min	Max	Distributed	Source
Cost per dollar					
Rituximab 50 mL:0.5 g/bottle	874.03	532.54	1,092.54	Gamma	a
Cyclophosphamide 0.2 g/branch	3.33	3.33	3.35	Gamma	a
Doxorubicin 10 mg/pill	3.10	2.97	3.18	Gamma	a
Vincristine 1 mg/branch	27.08	23.61	38.47	Gamma	a
Prednisone 5 mg/100 tablets	0.85	0.46	1.66	Gamma	a
Lenalidomide 25 mg/21 capsules	84.99	18.41	3,005.97	Gamma	a
Second-line cost	1,292.52	1,034.02	1,551.03	Gamma	a
Examination fee	144.29	115.43	173.15	Gamma	Zhang et al. (2020)
Adverse event handling costs					
Neutropenia	1,094.28	875.42	1,313.14	Gamma	Luo et al. (2022)
Thrombopenia	1,415.63	1,132.50	1,698.76	Gamma	Luo et al. (2022)
Anaemia	2,150.12	1,720.10	2,580.14	Gamma	Luo et al. (2022)
Adverse event rate					
Incidence of R-CHOP neutropenia	0.54	0.43	0.65	Beta	Nowakowski et al. (2021)
Incidence of R-CHOP thrombocytopenia	0.13	0.10	0.16	Beta	Nowakowski et al. (2021)
Incidence of R-CHOP anemia	0.20	0.16	0.24	Beta	Nowakowski et al. (2021)
Incidence of R2-CHOP neutropenia	0.60	0.48	0.72	Beta	Nowakowski et al. (2021)
Incidence of R2-CHOP thrombocytopenia	0.34	0.27	0.41	Beta	Nowakowski et al. (2021)
Incidence of R2-CHOP anemia	0.29	0.23	0.35	Beta	Nowakowski et al. (2021)
Utility					
PFS utility	0.83	0.66	0.99	Beta	Li et al. (2022)
PD utility	0.39	0.31	0.47	Beta	Li et al. (2022)
Discount rate	0.05	0.00	0.08-	-	Gan et al. (2023)

a represent median list price of a drug from a publicly available database.

This study initially employed the Get Data Graph Digitizer software to digitally extract survival rate data from the PFS and OS curves of the ECOG-ACRIN E1412 clinical trial. The SurvHE program package in R studio was utilized to model the extracted survival data. The choice of distribution was based on the goodness of fit measures from the Akaike Information Criteria (AIC) and Bayesian Information Criterion (BIC), with smaller the AIC and BIC values indicating a more appropriate distribution.

### Cost and utility data

From the perspective of Chinese healthcare system, this study considers direct medical costs, including drug purchase, patient examination, and adverse event management costs. Drug prices in China were sourced from the Pharmaceutical Database (https://db.yaozh.com/), reflecting the median of the latest winning bids from 2023 onwards. Adverse reaction incidences were obtained from clinical studies. The adverse reactions to be treated in this study were reduced neutrophil count, anemia, and decreased platelet count. The cost of test and treating adverse reactions were based on published literature (Luo et al., 2022; Zhang et al., 2020). To simplify the model, this study assumed the cost of treating adverse reactions as a one-time expense, without considering their impact on health utility value.

Since the referenced clinical trials did not report relevant utility values, the PFS and PD utility values for this study were taken from another published article on the cost-effectiveness of DLBCL in China, with PFS valued at 0.83 and PD at 0.39 (Li et al., 2022). The model derives its required periodic utility value from the annual utility value.

### Sensitivity analysis

The deterministic sensitivity analysis was performed to identify the impact of multiple uncertain factors on the results using the tornado diagram, with the specific parameter variation ranges detailed in Table 1. For undetermined parameters, ±20% of the baseline value is set as the upper and lower limits. Monte Carlo simulation was used in probabilistic sensitivity analysis to analyze parameters. The simulation was conducted 1,000 times. All cost distributions conformed to Gamma distribution, while all utility and percentage data followed the Beta distribution. The distribution statistics for each parameter conformed to normal distribution, with the standard deviation set at 10% of the mean.

### Scenario analysis

We assessed the sensitivity of different time horizons (10, 20, and 30 years) to the long-term survival outcomes of the model by scenario analysis. ICERs corresponding to time horizons were calculated and compared with WTP threshold to evaluate their cost-effectiveness.

### Subgroup analysis

We also conducted subgroup analysis to determine the cost-effectiveness in ABC subtype of DLBCL, which was classified as poor prognosis. The ECOG-ACRIN E1412 clinical trial included the analysis of the PFS and OS curves for two regimens treating ABC subtype of DLBCL. The methods for fitting survival curves and constructing models were consistent with the preliminary analysis. Data on costs and utility values referenced Table 1. Following the derivation of baseline results, sensitivity analysis was conducted.

## Results

### Base-case results

**FIGURE 2:**
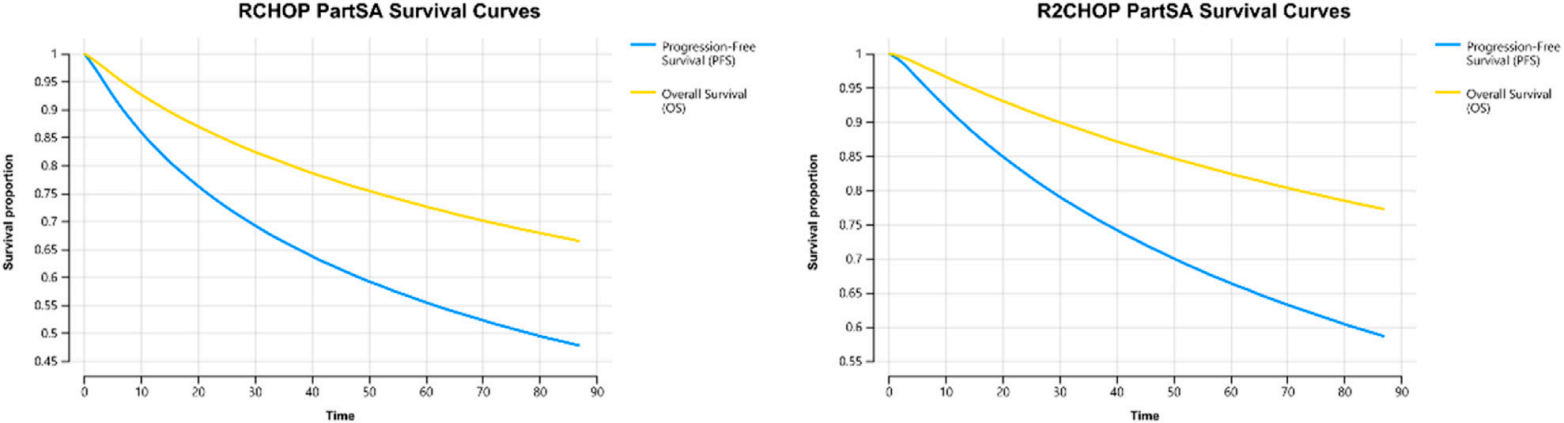
R-CHOP and R2-CHOP PartSA survival curves.

The parameters for distribution fitting are shown in Table 2, and the PFS and OS fitting curve for the two treatment regimens are shown in Figure 2. The results indicate that the lognormal distribution is the most suitable for data fitting. The basic analysis results of the partitioned survival model are shown in Table 3, detailing a total cost of $112,884.59 for the R2-CHOP group and $100,224.12 for the R-CHOP group. The R2-CHOP group achieved 3.42 QALYs, while R-CHOP group had 3.06 QALYs *per capita*. The cost-effectiveness ratio for R-CHOP is $35,159.06/QALY, falling below three times the *per capita* GDP ($37,232.5 per QALY).

**TABLE 2:** Results of fitting parameters of lognormal distribution.

Survival	Meanlog	Sdlog	AIC	BIC
PFS for R-CHOP	4.0	1.9	526.67	532.48
OS for R-CHOP	5.0	2.1	448.96	454.77
PFS for R2-CHOP	4.5	1.8	425.97	431.92
OS for R2-CHOP	5.6	2.0	344.33	350.29

**TABLE 3:** Base results of R2-CHOP versus R-CHOP.

	Cost	QALYs	Incr cost	Incr QALYs	ICER
R-CHOP	100,224.12	3.06			
R2-CHOP	112,884.59	3.42	12,660.47	0.36	35,159.06

### Sensitivity analysis

**FIGURE 3:**
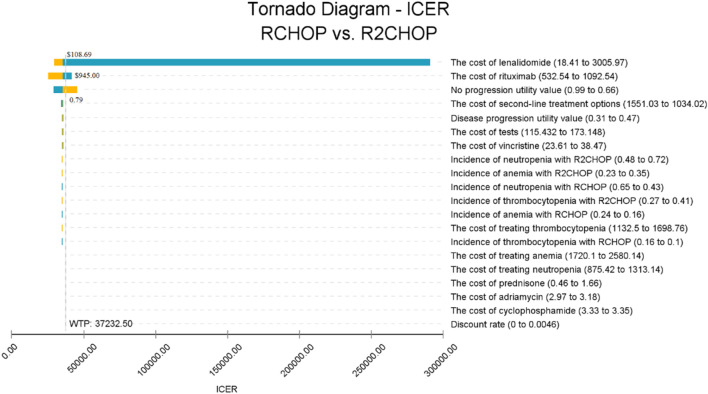
Tornado diagram of the deterministic sensitivity analysis.

**FIGURE 4:**
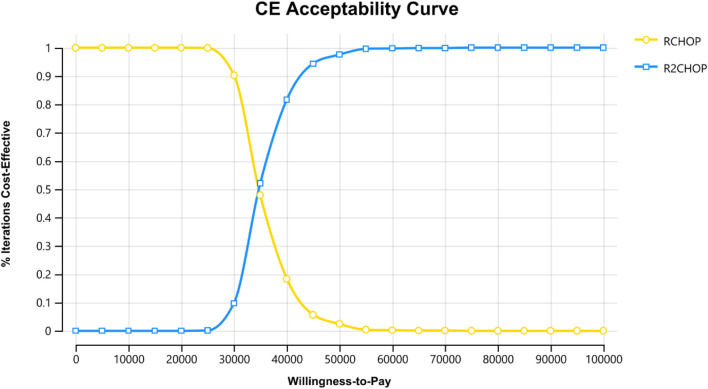
Cost-effectiveness acceptable curve (CEAC).

**FIGURE 5:**
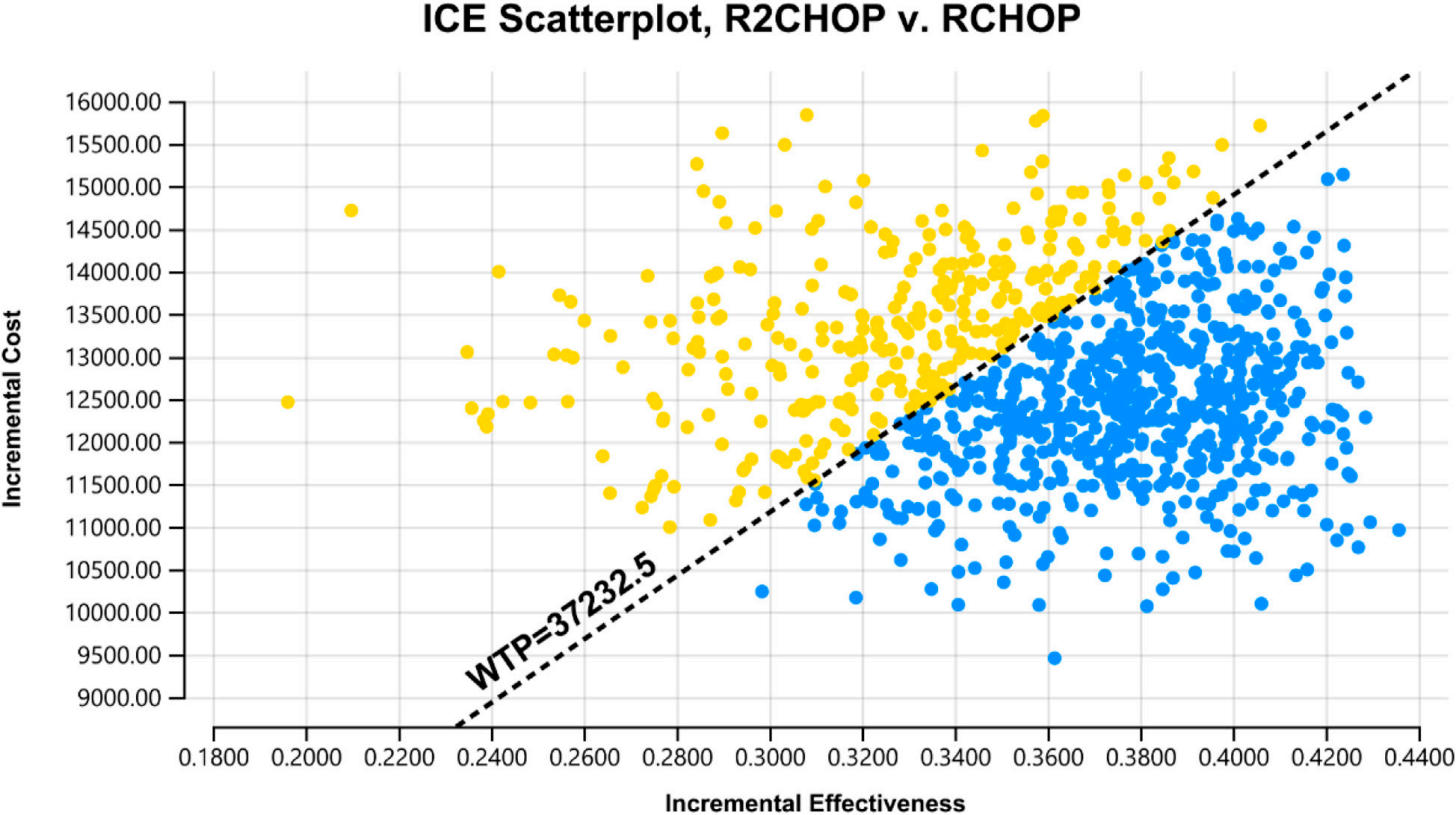
Scatter plot of probability sensitivity analysis.

Figure 3 reveals that the lenalidomide price, rituximab price, and PFS utility value are the three most influential factors, respectively. When the price of lenalidomide is $108.69, rituximab is $945.00 and that of PFS utility valued at 0.79, using three times the GDP *per capita* as the WTP value, R2-CHOP proves to be cost-effective. According to the acceptability curve and cost-effectiveness scatter plot (Figures 4, 5), R2-CHOP exhibits a 67.9% probability of being accepted over R-CHOP at the given threshold.

### Scenario analysis

In scenario analysis (Table 4), the time horizons were set to 10, 20, and 30 years, resulting in ICERs of $34,848.35/QALY, $36,063.28/QALY, and $37,175.93/QALY, respectively. The results indicate that the ICER for each time horizon remain below the established WTP threshold, suggesting that the treatment maintains favorable cost-effectiveness over longer time periods.

**TABLE 4:** Scenario analysis for different time horizons.

Time horizon	Incr cost	Incr QALYs	ICER
10 years	28,245.59	0.81	34,848.35
20 years	59,161.03	1.64	36,063.28
30 years	87,744.21	2.36	37,175.93

### Subgroup analysis

The model fitted to a curve using a lognormal distribution with a time horizon of 5 years shows that the total cost from patients with the ABC subtype of DLBCL received R2-CHOP treatment is $112,868.17 with a QALY of 3.31. The incremental cost is $15,970.50, with an incremental utility value of 0.49 QALYs and an ICER of $32,384.08/QALY, falling below three times the GDP *per capita*. At the set threshold, R2-CHOP exhibited 84.7% acceptability probability compared to R-CHOP.

## Discussion

The rising costs of cancer drugs pose a significant public health challenge by increasing financial burden on patients and healthcare systems worldwide (Vokinger, 2023). In China, chemotherapy remains the prevalent treatment for DLBCL. Following the November 2023 announcement of China’s ninth national centralized drug procurement batch, 266 products across 41 drug categories were selected. This selection spanned treatments for infection, tumor, cardiovascular, and cerebrovascular diseases, gastrointestinal diseases, mental diseases, chronic conditions, emergency medicine and drugs in short supply. These price reduction for the selected drugs is expected to significantly alleviate patient financial burdens. Lenalidomide capsules were promptly included in the procurement list after the patent expired. Therefore, in the context of Chinese healthcare, this study integrated lenalidomide as an oral anti-tumor agent into the standard R-CHOP regimen for DLBCL, revealing an increasing in both therapeutic effectiveness and cost. The analysis indicated that the cost-effectiveness of combination of lenalidomide and R-CHOP treatment surpassed that of the R-CHOP regimen, though the prices of lenalidomide and rituximab merit attention. Ashley’s study established a Markov model to assess the cost-effectiveness of R-CHOP and R2-CHOP for different subtypes of DLBCL in US. They found R-CHOP offered 5.2 QALYs at $69,920 while R2-CHOP delivered 6.5 QALYs for improved health outcomes at $143,753, making R2-CHOP’s ICER more favorable (Staton et al., 2015). The Qiushi’s study found that the R2-CHOP regimen might be highly cost-effective for DLBCL subtype-based therapies. It detailed the survival benefits and drug cost thresholds for novel agents, suggesting potential cost-effectiveness in subtype-based therapies by extensive parameters and model assumptions exploration (Chen et al., 2018).

Given the continuous introduction of new targeted drugs, it is essential to evaluate not just the cost-effectiveness of individual drugs, but also of combination chemotherapy regimens, to provide more choices for doctors and patients. This study involved various anti-tumor drugs, with single factor sensitivity analysis revealing lenalidomide as a primary factor influencing ICER. In China, the lenalidomide market has developed into a competitive arena between the original patent-holding company and several generic drug manufacturers. The Chinese government’s national centralized drug procurement policy has encouraged the substitution of domestic generic drugs for original brand drugs, effectively reducing drug prices and the financial burden on patients. Moreover, all generic drugs are required to pass the generic conformity assessment before they can be marketed (Yang et al., 2022). In 2024, the sale price for the original lenalidomide (Relfamide) in China is 21,643 yuan per box (25 mg/21 capsules), with the lowest sales price for lenalidomide capsules being 132.52 yuan (25 mg/21 capsules) from Changzhou Pharmaceutical Factory. The implementation of the ninth batch of national anthology drugs has led to a notable price difference between original and generic drugs, potentially influencing patients’ purchasing decision. Consequently, the R2-CHOP protocol may lose its cost-effectiveness if based on original drug prices. This indicates that China’s drug collection policy significantly affects the cost-effectiveness of lenalidomide prices when combined with R-CHOP regimens for DLBCL treatment. Similarly, lenalidomide drugs that are not competitively priced may also lack cost-effectiveness in combination with R-CHOP. The Lili’s study revealed a significant increase in the proportion of antihypertensive drugs included in the anthology in Chinese hospitals post-policy intervention, leading to a corresponding rise in the use of included drugs over those not included (Shang et al., 2024). Currently, a cost-effectiveness analysis comparing R-CHOP and R2-CHOP in China is lacking, and our study could offer valuable insights to pharmaceutical companies and healthcare policymakers.

There are also limitations in this study. Firstly, potential inaccuracies from using published Kaplan-Meier (KM) curves instead of individual patient data from the ECOG-ACRIN E1412 study for clinical data reconstruction. However, the KM curves were carefully recreated to ensure image-level accuracy. Secondly, the lack of utility value data for adverse events may result in inaccuracies in calculating QALYs. Thirdly, the applicability and generality of our findings to other countries might be constrained by variations in model inputs and research perspectives. In addition, we would like to consider the inclusion of a societal perspective in future studies, which would include indirect costs such as loss of productivity due to disease or disability, and other societal burdens associated with treatment, for a more comprehensive understanding of the cost-effectiveness of the R2-CHOP for the treatment of DLBCL.

## Conclusion

From the perspective of the Chinese healthcare system, combining lenalidomide with R-CHOP presents a cost-effective alternative to R-CHOP for treating DLBCL, including the ABC subtype. However, the price of lenalidomide and rituximab warrant attention.

## Data Availability

The raw data supporting the conclusions of this article will be made available by the authors, without undue reservation.
